# Molecular Characterization and Clinical Relevance of *ANXA1* in Gliomas *via* 1,018 Chinese Cohort Patients

**DOI:** 10.3389/fcell.2021.777182

**Published:** 2021-11-29

**Authors:** Zenghui Qian, Wenhua Fan, Fanlin Meng, Zhiyan Sun, Guanzhang Li, You Zhai, Yuanhao Chang, Changlin Yang, Fan Zeng, Ruichao Chai, Fan Wu, Zheng Zhao

**Affiliations:** ^1^ Beijing Tiantan Hospital, Capital Medical University, Beijing, China; ^2^ Beijing Neurosurgical Institute, Capital Medical University, Beijing, China; ^3^ CapitalBio Corporation, National Engineering Research Center for Beijing Biochip Technology, Beijing, China; ^4^ Chinese Glioma Genome Atlas Network, Beijing, China

**Keywords:** glioma, clinical behaviors, immune, macrophage, mesenchymal

## Abstract

Annexin A1 (ANXA1) is a calcium-dependent phospholipid-binding protein and has been implicated in multiple functions essential in cancer, including cell proliferation, apoptosis, chemosensitivity, metastasis, and invasion. However, the biological role and clinical behavior of *ANXA1* in glioma remain unclear. In this study, RNA-seq (*n* = 1018 cases) and whole-exome sequencing (WES) (*n* = 286 cases) data on a Chinese cohort, RNA-seq data with different histological regions of glioblastoma blocks (*n* = 270 cases), and scRNA-seq data (*n* = 7630 cells) were used. We used the R software to perform statistical calculations and graph rendering. We found that *ANXA1* is closely related to the malignant progression in gliomas. Meanwhile, *ANXA1* is significantly associated with clinical behavior. Furthermore, the mutational profile revealed that glioma subtypes classified by *ANXA1* expression showed distinct genetic features. Functional analyses suggest that *ANXA1* correlates with the immune-related function and cancer hallmark. At a single-cell level, we found that *ANXA1* is highly expressed in M2 macrophages and tumor cells of the mesenchymal subtype. Importantly, our result suggested that *ANXA1* expression is significant with the patient’s survival outcome. Our study revealed that *ANXA1* was closely related to immune response. *ANXA1* plays a key factor in M2 macrophages and MES tumor cells. Patients with lower *ANXA1* expression levels tended to experience improved survival. *ANXA1* may become a valuable factor for the diagnosis and treatment of gliomas in clinical practice.

## Introduction

Gliomas are the most common malignant brain tumor in adults. According to the 2016 WHO classification, glioma can be classified into five subtypes, namely, *IDH*-mutant lower-grade gliomas (LGGs) with chromosome 1p/19q co-deletion, *IDH*-mutant LGGs without 1p/19q co-deletion, *IDH* wild-type LGGs, *IDH*-mutant glioblastomas (GBMs), and *IDH* wild-type GBMs (Cancer Genome Atlas Research,N. et al., 2015; Louis et al., 2016). Although there have been advances of surgical resection followed by radiotherapy and chemotherapy with temozolomide (TMZ) in the past decades, patients with glioma still have poor prognosis, indicating that the main challenges underlying therapeutic failure are rooted in tumor heterogeneity (Jiang et al., 2016; Jiang et al., 2021). Studies of inter-tumor heterogeneity based on bulk tumor expression profiles found that GBMs exist in four subtypes, namely, proneural (TCGA-PN), classical (TCGA-CL), mesenchymal (TCGA-MES), and neural (TCGA-NE) (Verhaak et al., 2010). Recently, single-cell RNA-sequencing (scRNA-seq) has emerged as a critical technology to comprehensively depict the cellular states within tissues, both in health and in disease. By integrating single-cell RNA-sequencing (scRNA-seq) and other omics data, Neftel et al. found that malignant cells in GBMs exist in four cellular states that recapitulate 1) neural-progenitor–like (NPC-like), 2) oligodendrocyte-progenitor–like (OPC-like), 3) astrocyte-like (AC-like), and 4) mesenchymal-like (MES-like) states (Neftel et al., 2019), in which AC-like and MES-like cell types are enriched in TCGA-CL and TCGA-MES, and NPC- and OPC-like cell types are enriched in TCGA-PN. Although these findings shed much light on tumor heterogeneity, the relationships between the tumor and tumor microenvironment (TME) in glioma are still poorly understood.

As the first member of the annexin superfamily, annexin A1 (ANXA1) is a calcium-dependent phospholipid-binding protein. Previous studies suggest that loss of function or expression of this gene has been implicated in multiple functions essential in cancer, including cell proliferation, apoptosis, chemosensitivity, metastasis, and invasion (Bai et al., 2020; Feng et al., 2020; Xiong et al., 2021). Recently, Lin et al. investigated the prognostic and immune role of *ANXA1* in gliomas (Lin et al., 2021). However, the systematic and comprehensive transcriptome characterization of *ANXA1* in gliomas is unclear. In this study, we integrated bulk genomic and transcriptomic profiles and scRNA-seq data to comprehensively characterize *ANXA1*’s role in gliomas. Our work provides an insight on *ANXA1*’s role in glioma, which might translate to clinical application for future diagnosis and therapy in glioma.

## Materials and Methods

### Patients and Samples

All RNA-sequencing data of diffuse glioma patients were obtained from two independent databases: the CGGA dataset (**Dataset 1**, *n* = 325 cases) (http://www.cgga.org.cn) and the CGGA dataset (**Dataset 2**, *n* = 693 cases) (Zhao et al., 2021). To compare the gene expression patterns of tumor tissues and normal brain tissues, we also collected 20 RNA-seq samples of normal brain tissues from the CGGA database in this study. All WES data of diffuse glioma patients from WHO II-IV were obtained from the CGGA Network (**Dataset 3**, *n* = 286). Clinical information of all patients was also collected from the CGGA Network, such as WHO grade (WHO II-IV), histology grade (oligodendroglioma, anaplastic oligodendroglioma, astrocytoma, anaplastic astrocytoma, and glioblastoma, abbreviated as O, AO, A, AA, and GBMs, respectively); gender, age, and overall survival data; progression status (primary and recurrent states); and molecular pathological features (*IDH* mutation status, *MGMT* promoter methylation status, and chromosome 1p/19q co-deletion status). This research was approved by the Ethics Committee of Capital Medical University, and all patients provided written informed consent.

To further explore *ANXA1* expression in different histological regions of GBM blocks, we obtained Ivy data from the Ivy Glioblastoma Atlas Project–Allen Institute for Brain Science datasets (**Dataset 4,** *n* = 270 cases) (Puchalski et al., 2018) (http://glioblastoma.alleninstitute.org/). For this dataset, we collected different histological regions that contain 1) cellular tumor (CT), 2) infiltrating tumor (IT), 3) leading edge (LE), 4) microvascular proliferation (MP), and 5) pseudopalisading cells (PC).

The scRNA-seq data of diffuse glioma patients were obtained from a previous study (Neftel et al., 2019) (https://singlecell.broadinstitute.org/single_cell/study/SCP393/single-cell-rna-seq-of-adult-and-pediatric-glioblastoma). Of them, there are 6863 tumor cells, 754 macrophages, 219 oligodendrocytes, and 94 T cells (**Dataset 5**). For tumor cells, we also obtained four types of cellular state annotations that recapitulate 1) neural-progenitor–like (NPC-like), 2) oligodendrocyte-progenitor–like (OPC-like), 3) astrocyte-like (AC-like), and 4) mesenchymal-like (MES-like) states.

### CGGA CNV Data Analysis

WES data were mapped to the human reference genome (hg19) using the Burrows–Wheeler Aligner (BWA) tool (Li and Durbin, 2009) with default parameters. Then, SAMtools (Li et al., 2009) and Picard (http://broadinstitute.github.io/picard/) were used to sort the reads by coordinates and mark duplicates. Next, we used the CNVkit software (Talevich et al., 2016) to estimate the CNA status of well-known driver genes in gliomas, such as *PTEN*, *MET*, *EGFR*, and *CDKN2A/B*. In this study, a copy number gain is identified as log2 (ratio) larger than 0.5, while a copy number loss is identified as log2 (ratio) less than 1.0.

### Immune Proportion Analysis

For RNA-seq data, we estimated the abundance of member cell types using the CIBERSORT method (Newman et al., 2015). We uploaded gene expression profiles and ran CIBERSORT software online (https://cibersort.stanford.edu/runcibersort.php) by selecting LM22 (gene signature) and 1000 permutation parameters. As result, we obtained the 22 kinds of cell composition for each sample from gene expression profiles.

### TCGA Molecular Classifications for Each Sample

For RNA-seq data, we identified the TCGA subtypes for each sample as previously described (Wang et al., 2017). In this pipeline, ssGSEA was performed to obtain the scores of the four signatures for each sample from gene expression profiles. Since the scores of the four signatures were not directly comparable, this pipeline was used to perform a resampling procedure to generate null distributions for each of the four subtypes (1000 permutations). Following this procedure, this method generated random ssGSEA scores for each subtype to provide empirical *p-*values and scaled ssGSEA scores for the raw ssGSEA scores of each sample. Finally, we assigned the TCGA subtypes for each sample based on the *p-*values and scaled ssGSEA scores.

### Immunohistochemistry Analysis

The selected glioma samples were collected from the CGGA tissue bank and were supervised by the Beijing Tiantan Hospital Institutional Review Board (KY 2019-143-02). IHC analysis was performed as previously reported (Hu et al., 2018). Briefly, the slides were deparaffinized and boiled in antigen-retrieval buffer. Then, the slides were blocked using endogenous peroxidase with H_2_O_2_, subsequently blocking non-special sites, and the slides were incubated with primary antibodies against ANXA1 (Cell Signaling Technology #32934, 1:400 dilution) overnight at 4°C. On the second day, the slides were rinsed three times in PBS buffer and incubated with the secondary antibody working solution (PV6000 Beijing Zhongshan Jinqiao Biological Company) for 60 min at room temperature. Last, the IHC images were captured using an Axio Imager 2 microscope (Zeiss). The scores were calculated according to the intensity score multiplied by the areas as follows: The intensity was defined as follows: 0 for no staining, one for weak staining, two for moderate staining, and three for strong staining. The area score was determined as follows: 0 for less than 5% cells positive, 1 for 5–25% cells positive, 2 for 26–50% cells positive, 3 for 51–75% cells positive, and 4 for greater than 75% cells positive.

### Gene Set Enrichment Analysis

To investigate the biological functions of the *ANXA1* gene, the *ANXA1* coexpressed genes were obtained and gene set enrichment analysis (GSEA) (Subramanian et al., 2005) was performed. First, we downloaded the gene sets from the GSEA website (http://www.gsea-msigdb.org/gsea), including the Gene Ontology (GO) biological process, Kyoto Encyclopedia of Genes and Genomes (KEGG), and cancer hallmark. Then, *ANXA1* coexpressed genes were obtained by the Pearson expression correlation analysis between *ANXA1* and other genes. Finally, we implemented the ClusterProfiler R package to reach this process (Yu et al., 2012).

### Statistical Analysis

The R statistical software (v4.0.3) (http://www.r-project.org) was used for statistical calculations and graph rendering. The prognostic value of *ANXA1* was estimated by using the Kaplan–Meier analysis and Cox proportional hazard model analysis using the “survival” and “survminer” packages in R. In this study, the Pearson correlation analysis was used to obtain *ANXA1* coexpressed genes. In particular, a positive correlation is defined as a correlation coefficient larger than 0.6 and *p*-value < 0.05, while a negative correlation is defined as a correlation coefficient less than −0.6 and *p*-value < 0.05. The Wilcoxon test and one-way ANOVA test were used for two and multiple group comparisons, respectively. For all statistical methods, *p* < 0.05 was considered as a significant difference.

## Results

### Patient Characteristics

In this study, a total of 1,018 patients with gliomas aged 8–79 years (median ± sd, 42 ± 12 years) were included. The majority of glioma patients were males (59%) and WHO IV (38%), and there were 651 cases of primary gliomas. For these patients, 617 case deaths were recorded, with the median survival of NA (3470-NA), 1208 (1028-1657), and 378 (344-415) for WHO II, WHO III, and WHO IV, respectively. All patients with transcriptomic data were used to analyze *ANXA1* expression, and 231 of patients were also performed with WES to investigate genetic changes. The clinical and pathological features of these patients are described in Table 1.

**TABLE 1:** Clinical characteristics of the sample set according to *ANXA1* expression status.

Characteristic	CGGA_325			CGGA_693		
	Total (N = 325)	*ANXA1* high (N = 163)	*ANXA1* low (N = 162)	Total (N = 693)	*ANXA1* high (N = 347)	*ANXA1* low (N = 346)
PRS type (%)						
Primary	229 (70.5)	104 (63.8)	125 (77.2)	422(60.9)	175 (50.4)	247 (71.4)
Recurrent	92 (28.3)	55 (33.7)	37 (22.8)	271 (39.1)	172 (49.6)	99 (28.6)
Unknown	4 (1.2)	4 (2.5)	0 (0.0)	0 (0.0)	0 (0.0)	0 (0.0)
Grade (%)						
WHO II	103 (31.7)	12 (7.4)	91 (56.2)	188 (27.1)	58 (16.7)	130 (37.6)
WHO III	79 (24.3)	39 (23.9)	40 (24.7)	255 (36.8)	100 (28.8)	155 (44.8)
WHO IV	139 (42.8)	108 (66.3)	31 (19.1)	249 (35.9)	188 (54.2)	61 (17.6)
Unknown	4 (1.2)	4 (2.5)	0 (0.0)	1 (0.1)	1 (0.3)	0 (0.0)
Histology (%)						
Astrocytoma	56 (17.2)	13 (8.0)	43 (26.5)	119 (17.2)	48 (13.8)	71 (20.5)
Anaplastic astrocytoma	62 (19.1)	38 (23.3)	24 (14.8)	152 (21.9)	78 (22.5)	74 (21.4)
Anaplastic oligodendroglioma	12 (3.7)	0 (0.0)	12 (7.4)	82 (11.8)	22 (6.3)	60 (17.3)
Anaplastic oligoastrocytoma	0 (0.0)	0 (0.0)	0 (0.0)	21 (3.0)	0 (0.0)	21 (6.1)
Glioblastoma	139 (42.8)	108 (66.3)	31 (19.1)	249 (35.9)	188 (54.2)	61 (17.6)
Oligodendroglioma	52 (16.0)	0 (0.0)	52 (32.1)	60 (8.7)	10 (2.9)	50 (14.5)
Oligoastrocytoma	0 (0.0)	0 (0.0)	0 (0.0)	9 (1.3)	0 (0.0)	9 (2.6)
Unknown	0 (0.0)	2 (1.8)	2 (0.9)	1 (0.1)	1 (0.3)	0 (0.0)
Age (years)						
Mean ± sd	42.9 ± 11.96	46.7 ± 12.74	39.1 ± 9.74	43.2 ± 12.39	44.9 ± 13.38	41.7 ± 11.10
Gender (%)						
Male	203 (62.5)	106 (65.0)	97 (59.9)	398 (57.4)	206 (59.4)	192 (55.5)
*IDH* mutation (%)						
Mutation	175 (53.8)	40 (24.5)	135 (83.3)	356 (51.4)	134 (38.6)	222 (64.2)
Wild type	149 (45.8)	123 (75.5)	26 (16.0)	286 (41.3)	208 (59.9)	78 (22.5)
Unknown	1 (0.3)	0 (0.0)	1 (0.6)	51 (7.4)	5 (1.4)	46 (13.3)
1p/19q co-deletion status (%)						
Co-deletion	67 (20.6)	3 (1.8)	64 (39.5)	145 (20.9)	30 (8.6)	115 (33.2)
Non–co-deletion	250 (76.9)	155 (95.1)	95 (58.6)	478 (69.0)	315 (90.8)	163 (47.1)
Unknown	8 (2.5)	5 (3.1)	3 (1.9)	70 (10.1)	2 (0.6)	68 (19.7)
*MGMT* promoter methylation status (%)						
Methylated	157 (48.3)	69 (42.3)	88 (54.3)	315 (45.5)	154 (44.4)	161 (46.5)
Un-methylated	149 (45.8)	86 (52.8)	63 (38.9)	227 (32.8)	120 (34.6)	107 (30.9)
Unknown	19 (5.8)	8 (4.9)	11 (6.8)	151 (21.8)	73 (21.0)	78 (22.5)
TCGA subtype (%)						
CL	71 (21.8)	68 (41.7)	3 (1.9)	140 (20.2)	103 (29.7)	37 (10.7)
MES	75 (23.1)	70 (42.9)	5 (3.1)	143 (20.6)	121 (34.9)	22 (6.4)
NE	44 (13.5)	8 (4.9)	36 (22.2)	132 (19.0)	35 (10.1)	97 (28.0)
PN	135 (41.5)	17 (10.4)	118 (72.8)	278 (40.1)	88 (25.4)	190 (54.9)
Radiotherapy status (%)						
Therapy	244 (75.1)	116 (71.2)	128 (79.0)	510 (73.6)	261 (75.2)	249 (72.0)
Without therapy	66 (20.3)	37 (22.7)	29 (17.9)	136 (19.6)	59 (17.0)	77 (22.3)
Unknown	15 (4.6)	10 (6.1)	5 (3.1)	47 (6.8)	27 (7.8)	20 (5.8)
Chemotherapy status (%)						
Therapy	193 (59.4)	105 (64.4)	88 (54.3)	486 (70.1)	264 (76.1)	222 (64.2)
Without therapy	111 (34.2)	48 (29.4)	63 (38.9)	161 (23.2)	61 (17.6)	100 (28.9)
Unknown	21 (6.5)	10 (6.1)	11 (6.8)	46 (6.6)	22 (6.3)	24 (6.9)

### ANXA1 Is Associated With Malignant Progression of Gliomas

**FIGURE 1:**
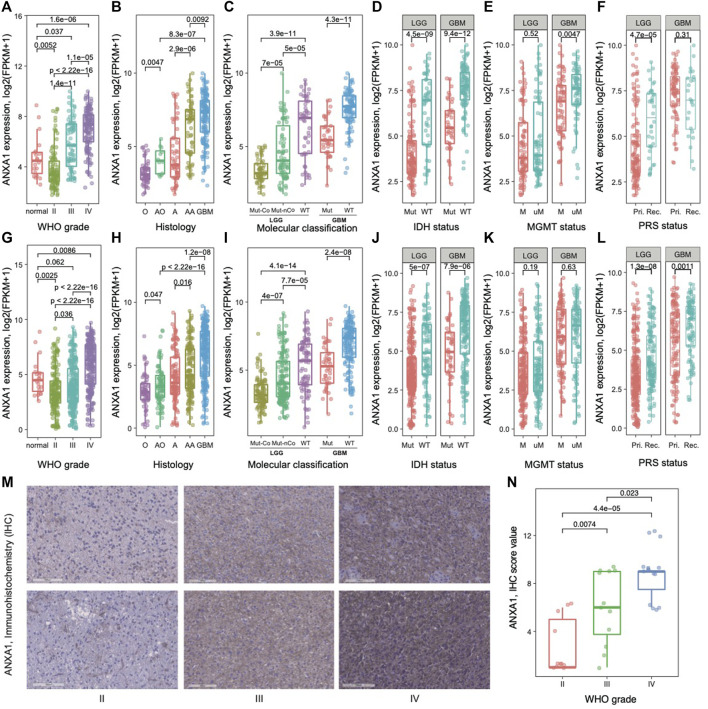
Gene expression pattern of *ANXA1* in glioma. **(A)** and **(G)** Normal brain and WHO II–IV; **(B)** and **(H)** histology; **(C)** and **(I)** 2016 WHO classification; **(D)** and **(J)** IDH mutation status in LGGs and GBMs; **(E)** and **(K)** MGMT promoter methylation status in LGGs and GBMs; **(F)** and **(L)** primary (Pri.)/recurrent (Rec.) status in LGGs and GBMs. **(A–F)** for **Dataset 1** and **(G–L)** for **Dataset 2**. **(M)** Representative immunohistochemistry (IHC) staining of *ANXA1* in different grades of gliomas. **(N)** Comparing *ANXA1* expression in gliomas with WHO II–IV by Dot plots.

To explore *ANXA1*’s role in gliomas, we examined its transcriptomic level in different subtypes of gliomas in two batches of RNA-seq data from the CGGA database. We found that the expression values of *ANXA1* were significantly higher in GBM patients than in those with normal brain and lower-grade gliomas (WHO II and WHO III) in **Dataset 1** (*p* < 5e-5, Figure 1A). Our further results showed that the *ANXA1* expression levels were statistically more abundant in GBMs than in other histology (*p* < 1e-2, Figure 1B). In addition, due to the genetic and clinical differences between *IDH-*mutated gliomas and *IDH* wild-type gliomas, we explored the role *ANXA1* played in gliomas with different *IDH* statuses. The *ANXA1* expression was highest in *IDH* wild-type and lowest in *IDH* mutation and 1p/19q co-deletion in LGGs (all *p* ≤ 5e-5, Figure 1C left), while *ANXA1* expression was higher in the *IDH* wild-type than in *IDH* mutant gliomas in GBMs (*p* < 5e-11, Figure 1C right). There was a reduced expression of *ANXA1* in glioma with *IDH* mutation based on LGGs and GBMs (all *p* < 5e-9, Figure 1D). It is well known that the *MGMT* promoter methylation status is a key biomarker indicating temozolomide (TMZ) chemotherapy sensitivity in gliomas. As a result, we found that patients without *MGMT* promoter methylation possessed a higher *ANXA1* expression level in GBMs, suggesting that *ANXA1* may play a resistance role in TMZ therapy of GBMs (*p* < 5e-3, Figure 1E). Notably, we also found that *ANXA1* expression was higher in recurrent LGGs (Figure 1F). The aforementioned results are well validated in independent CGGA RNA-seq data (Figures 1G–L). Consistently, the immunohistochemistry (IHC) experiments of glioma patients (WHO II–IV grade) showed that *ANXA1* was the highest in WHO IV patients and lowest in WHO II patients (all *p* < 0.05, Figures 1M–N). Taken together, these results suggest that the *ANXA1* gene acts as an oncogene and may serve as a biomarker for disease progression in gliomas.

### ANXA1 Clinicopathological Features of Glioma Specimens

**FIGURE 2:**
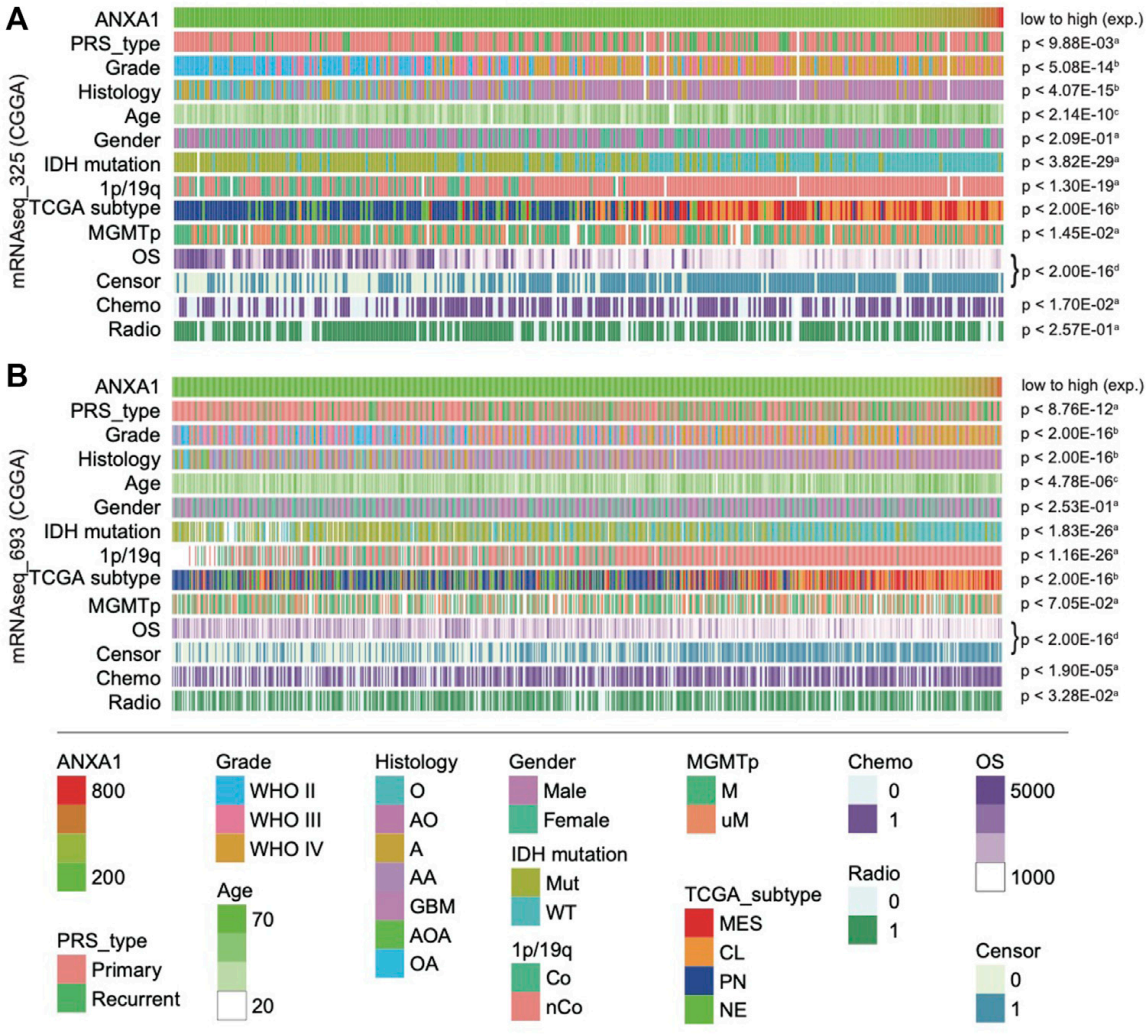
Landscape of clinical and molecular characteristics associated with *ANXA1* expression in gliomas. **Dataset 1** **(A)** and **Dataset 1** **(B)** were arranged in an increasing order of *ANXA1* expression. The relationship between *ANXA1* expression and patients’ characteristics was evaluated: (a, Wilcoxon rank sum tests between two groups; b, one-way ANOVA between several groups; c, Spearman’s correlation tests between *ANXA1* expression and continuous variables; d, Log-rank test for survival data).

To investigate the clinical value of *ANXA1*, we examined the association between gene expression of *ANXA1* and clinical information, including primary/recurrent status, WHO grade, histology, age, gender, well-known molecular status, TCGA subtype, survival, and therapy information. The evaluation of the association between clinicopathological features and the *ANXA1* gene was conducted for 1,018 glioma patients from a Chinese cohort. Gliomas in **Dataset 1** were ordered by increasing *ANXA1* expression (Figure 2A). Our results showed that primary gliomas and LGGs had lower levels of *ANXA1* expression (all *p* < 0.01), suggesting that *ANXA1* may play a positive role in malignant progression. Younger patients with glioma had lower expression of *ANXA1* (*p* < 2.14e-10). Gender of patients is not associated with *ANXA1* expression. With regard to genomic alterations, *IDH* mutation, 1p/19q co-deletion, and *MGMT* promoter methylation indicated lower *ANXA1* expression (all *p* < 0.01). Gliomas with lower *ANXA1* expression are more likely to belong to proneural (PN) and neural (NE) subtypes and have a good prognosis, while gliomas with high *ANXA1* expression are more likely to belong to mesenchymal (MES) and classical (CL) subtypes and have poor survival (all *p* < 2.00e-16). Gliomas with chemotherapy and/or radiotherapy tend to have a high *ANXA1* expression. The aforementioned results are well validated in independent CGGA RNA-seq data (**Dataset 2,** Figure 2B). These results indicate that *ANXA1* is closely related to clinical behavior.

### Genomic Features of ANXA1 Expression Subtypes in Gliomas

To investigate the association between *ANXA1* expression and genomic alterations, we analyzed the somatic mutations and copy number alteration data from cases with RNA-seq and WES data for this purpose. In total, 231 samples in the entire cohort harbored both RNA-seq and WES data (**Dataset 3**). Recapitulating previous studies, we confirmed frequency mutation in *IDH*, *TP53*, *ATRX*, *CIC*, *NOTCH1*, *EGFR*, and *PDGFRA* in this study. According to *ANXA1* expression, gliomas were divided into G1 group (low expression, *n* = 116) and G2 group (high expression, *n* = 115). Approximately two-thirds of cases in the G1 group carried either an *IDH1* mutation or *IDH2* mutation.

**FIGURE 3:**
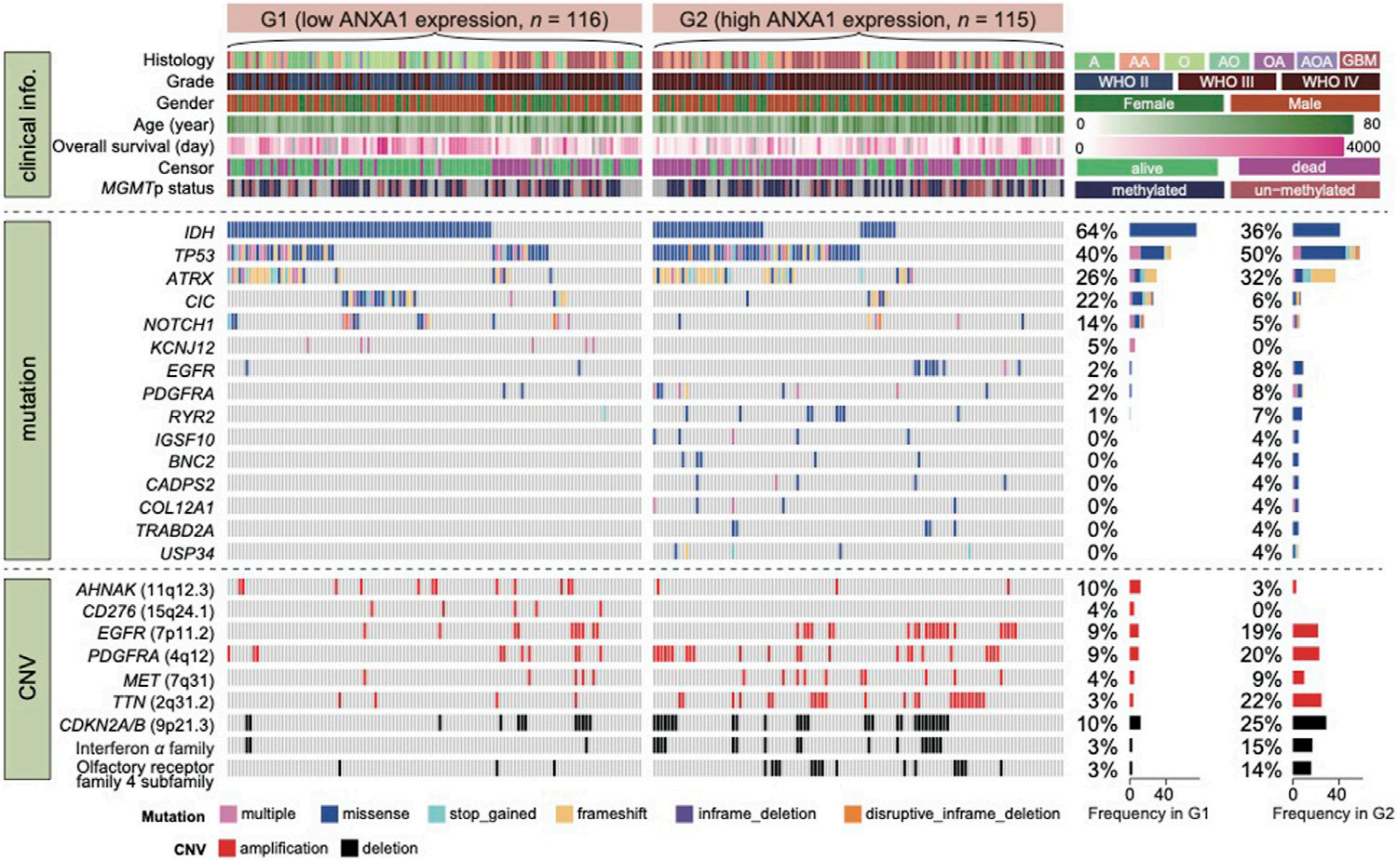
Mutational landscape of glioma with high and low expression of *ANXA1*. **Dataset 3** was classified in two groups according to *ANXA1* expression. Alterations in common driver and novel genes are displayed. Cases with both RNA-seq and WES data (*n* = 231) were enrolled for this analysis.

In addition, cases in the G1 group were enriched in *CIC* and *NOTCH1* mutation that have been well-described in oligodendroglia histology (Figure 3). In contrast, both *TP53* and *ATRX* mutation in cases of the G2 group were 1.25 times higher than those in the G1 group. On the other hand, cases in the G2 group have a much higher mutation frequency of *EGFR* and *PDGFRA* than those in the G1 group. Notably, although previously not recognized, mutations in *RYR2*, *IGSF10*, *BNC2*, *CADPS2*, *COL12A1*, *TRABD2A*, and *USP34* were significantly enriched in the G2 group. Moreover, we also explored the frequency of copy number alterations in G1 and G2 groups. For CN amplification, G1 had a higher alteration frequency in *AHNAK* and *CD276*, while G2 had a high alteration frequency in *EGFR*, *PDGFRA*, *MET*, and *TTN*. For CN loss, our results showed that deletion in *CDKNA2A/B* genes in interferon-*α* family and olfactory receptor family 4 subfamilies mainly occurred in G2 cases. Taken together, glioma subtypes classified by *ANXA1* expression showed distinct mutation and CNA features.

### ANXA1 Correlates With Immune-Related Function and Cancer Hallmark in Glioma Ecosystem

**FIGURE 4:**
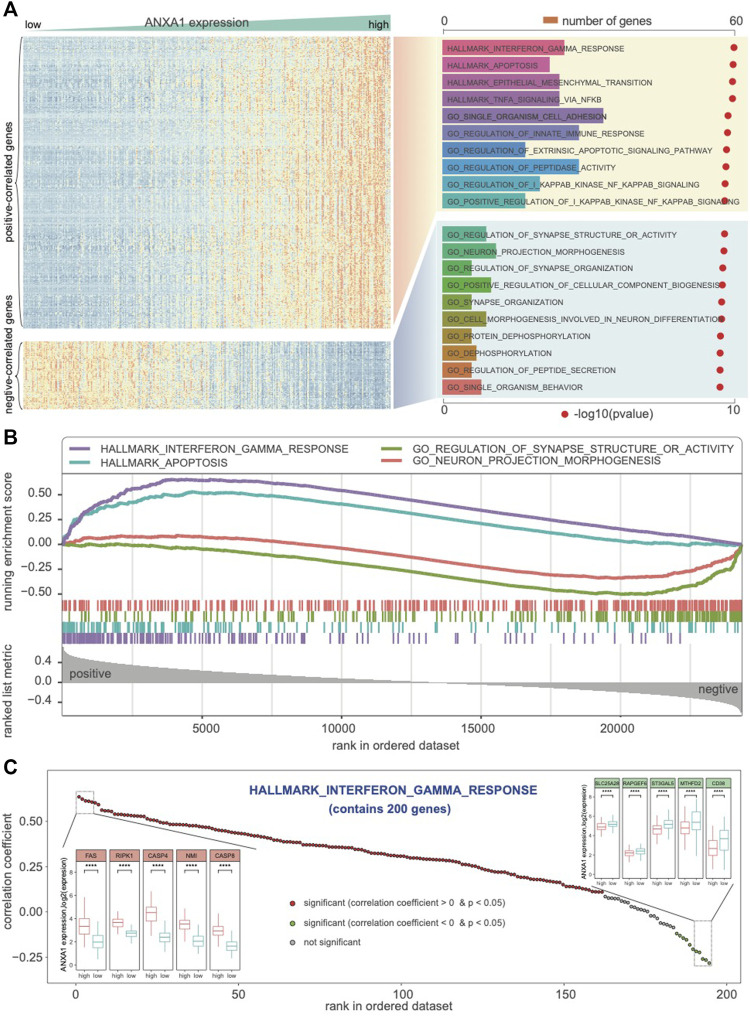
*ANXA1* involved in the biological process and cancer hallmark. **(A)** Biological functions related to immune response, interferon-gamma response, and regulation of innate immune response were significantly positively correlated with *ANXA1* expression (R > 0.6 and *p* < 0.05). **(B)** GSEA indicated that *ANXA1* was significantly associated with immune phenotypes and neuro-associated function. **(C)** *ANXA1* was significantly correlated with the genes in the hallmark of interferon-gamma response.

*ANXA1* expression was heterogeneous in different glioma subtypes. To explore *ANXA1*’s biological role in gliomas, RNA-seq data were collected. First, we obtained the genes that significantly correlated with *ANXA1* expression (Pearson |*R*| > 0.6 and *p* < 0.05). Totally, 462 positive and 107 negative coexpressed genes were identified in **Dataset 1**. Then, we predicted the GO biological process and cancer hallmark of these coexpressed genes. GSEA showed that the coexpressed positive genes associated with *ANXA1* were mainly involved in immune-related functions, such as interferon-gamma response and regulation of innate immune response, suggesting a regulatory role in the immune microenvironment in gliomas (Figure 4A). In particular, we found that these coexpressed genes also positively function in apoptosis, epithelial–mesenchymal transition, NF-κB signaling, etc., indicating that *ANXA1* may play an important role in regulating cell fate in gliomas. In contrast, we found that coexpressed negative genes of *ANXA1* participate in the neuro-basic functions in gliomas, such as synapse structure and organization, regulation of cellular component biogenesis, and neuro-projection morphogenesis and differentiation. Furthermore, GSEA verified that *ANXA1* was associated with immune, apoptosis, and neuron function (Figure 4B). For genes in interferon-gamma response, we confirmed that they are associated with *ANXA1* expression, and show the differential expressed patterns in glioma subtypes grouped by *ANXA1* expression (Figure 4C). In summary, *ANXA1* correlates with immune-related function and cancer hallmark and plays a critical role in the glioma ecosystem.

### ANXA1 Is Highly Expressed in M2 Macrophages and MES Tumor Cells

**FIGURE 5:**
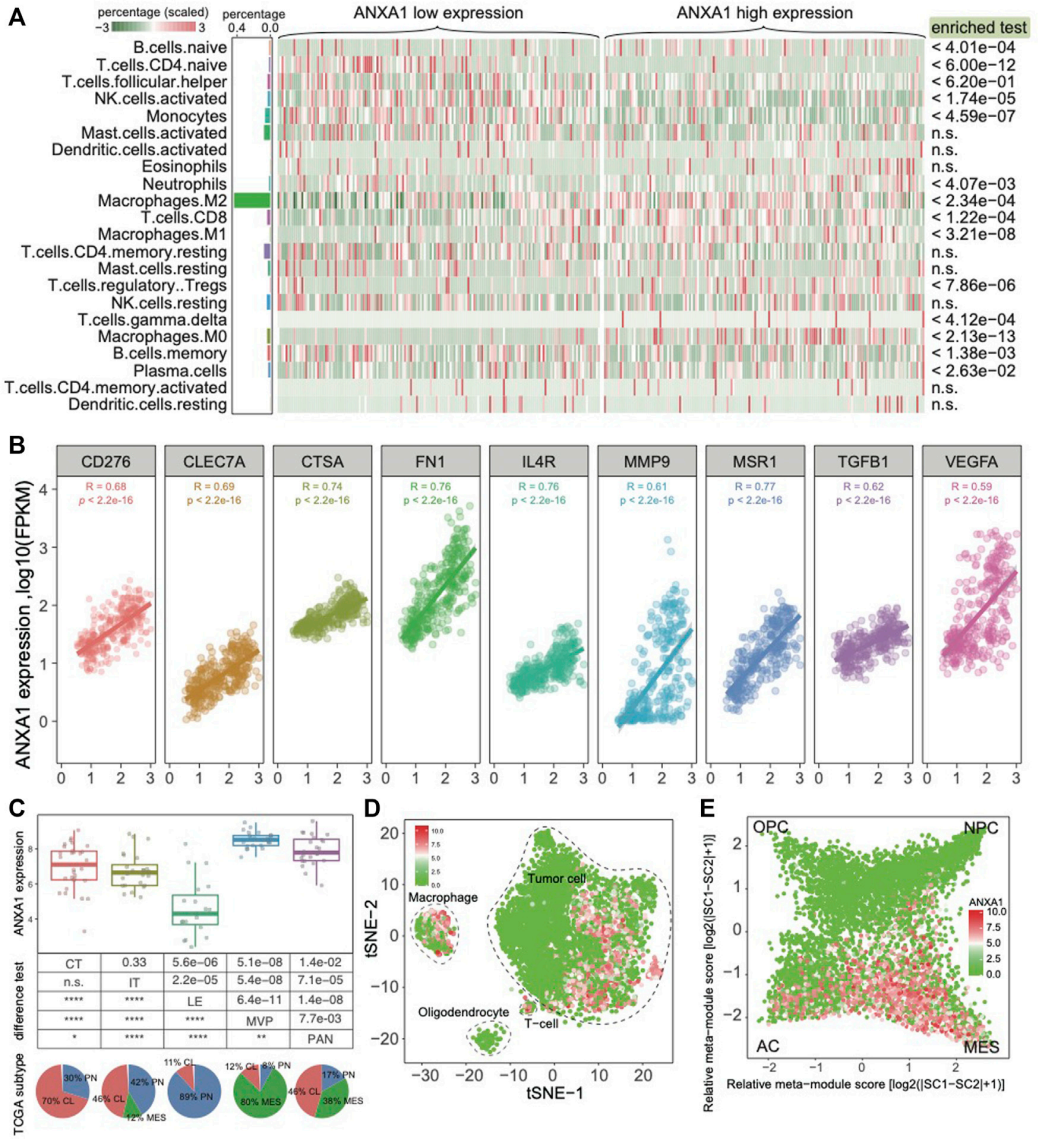
*ANXA1* highly expressed in M2 macrophages and MES tumor cells. **(A)** Cell component of gliomas grouped by *ANXA1* expression. **(B)** *ANXA1* expression positively associated markers of M2 macrophages. **(C)** Expression pattern of *ANXA1* in different histological regions of GBM blocks. **(D)** The single-cell data showed that *ANXA1* was mainly expressed in tumor cells and macrophages. **(E)** Expression pattern of *ANXA1* in the glioma cellular state.

As *ANXA1* confers an extended immune status, we sought to further explore the *ANXA1* regulatory immune role in the glioma ecosystem. We applied CIBERSORT software on **Dataset 1** for estimating the relative abundances of 22 infiltrating immune cells (Newman et al., 2015). These cells mainly include lymphocytes, plasma, myeloid cells, and eosinophils. As a result, the majority of cell types in gliomas are myeloid cells and lymphocytes. In addition, we found that M2 macrophages are significantly enriched in gliomas with high *ANXA1* expression (Figure 5A). We also validated that *ANXA1* expression exhibited a significant positive correlation with the expression of M2-related genes (all *R* > 0.6 and *p* < 2.2e-16), including *CD276*, *CLE7A*, *CTSA*, *FN1*, *IL4R*, *MMP9*, *MSR1*, *TGFB1*, and *VEGFA* (Figure 5B), suggesting that *ANXA1* acts a potential regulatory factor for M2 macrophages. In addition, we further collected anatomic transcriptional data in gliomas (**Dataset 4**), including leading edge (LE), infiltrating tumor (IT), cellular tumor (CT), pseudopalisading cells around necrosis (PAN), and microvascular proliferation (MVP) (Puchalski et al., 2018). Therefore, we found that *ANXA1* was significantly under-expressed in CL enriched in the PN TCGA subtype and significantly overexpressed in MVP enriched in the MES TCGA subtype. This result is consistent with previous findings that *ANXA1* was highly expressed in MES gliomas. To further explore *ANXA1*’s role in the tumor environment, we collected single-cell transcriptomic data in gliomas from a previous study (Neftel et al., 2019) (**Dataset 5**). We found that *ANXA1* is highly expressed in macrophages, indicating a potential role for macrophages, especially M2 macrophages (Figure 5D). Moreover, we also noticed that tumor cells are highly expressed in the *ANXA1* gene. In the single-cell level, our result showed that tumor cells with high expression of *ANXA1* are in the MES cellular state (Figure 5E), indicating that *ANXA1* could drive transitions to MES-like states in gliomas as reported in a previous study (Hara et al., 2021). In summary, we found that *ANXA1* is highly expressed in M2 macrophages and MES tumor cells.

### ANXA1 Is a Prognostic Model for Predicting OS in Gliomas

**FIGURE 6:**
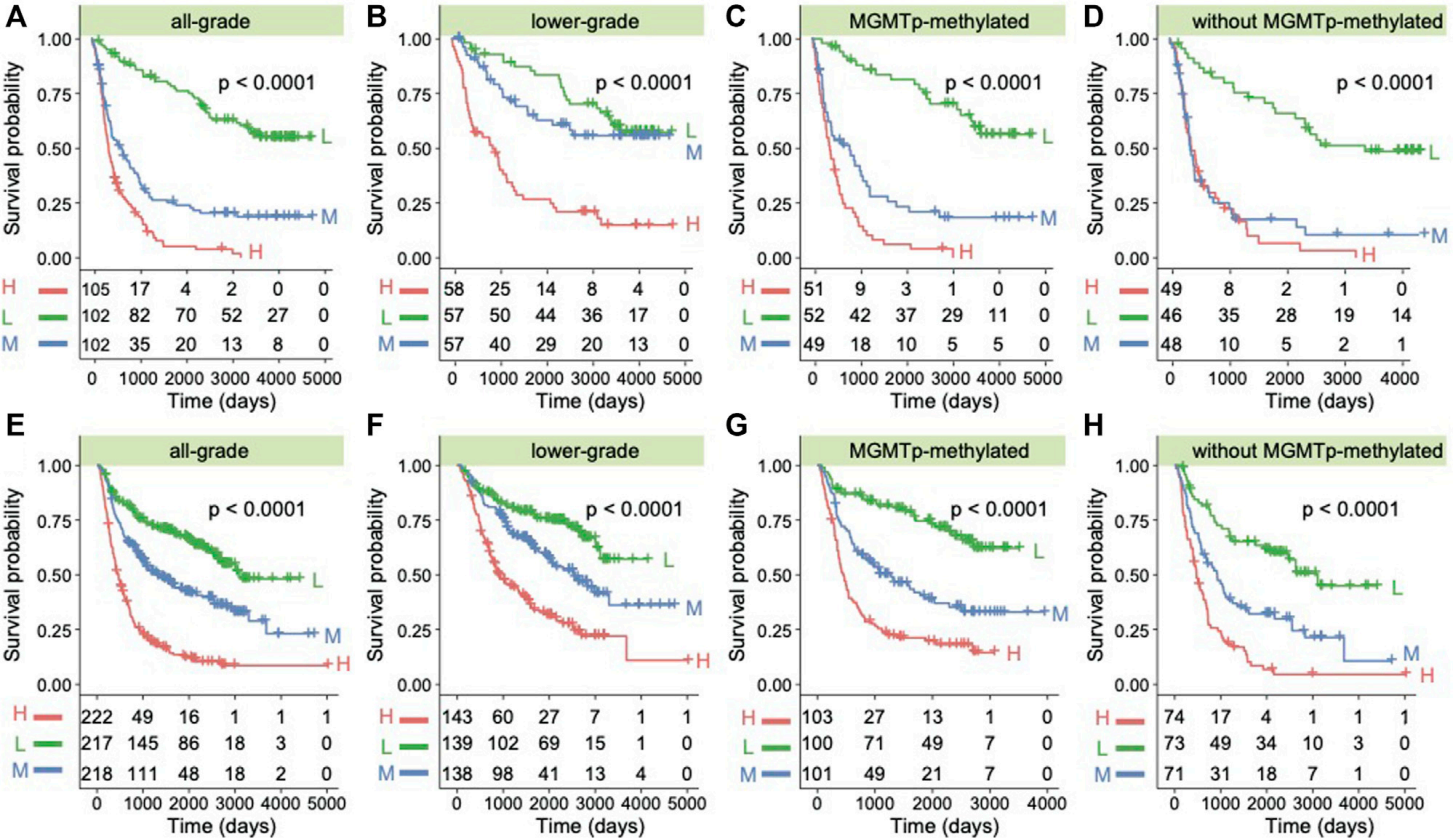
*ANXA1* was a prognostic factor in glioma patients. **(A)** Kaplan–Meier survival analysis of all grades of glioma patients in **Dataset 1** based on *ANXA1* expression. **(B)** Kaplan–Meier survival analysis of LGG patients in **Dataset 1** based on *ANXA1* expression. **(C)** Kaplan–Meier survival analysis of patients with MGMT promoter methylation in **Dataset 1** based on *ANXA1* expression. **(D)** Kaplan–Meier survival analysis of patients without MGMT promoter methylation in **Dataset 1** based on *ANXA1* expression. **(E)** Kaplan–Meier survival analysis of all grades of glioma patients in **Dataset 2** based on *ANXA1* expression. **(F)** Kaplan–Meier survival analysis of LGG patients in **Dataset 2** based on *ANXA1* expression. **(G)** Kaplan–Meier survival analysis of patients with MGMT promoter methylation in **Dataset 2** based on *ANXA1* expression. **(H)** Kaplan–Meier survival analysis of patients without MGMT promoter methylation in **Dataset 2** based on *ANXA1* expression.

To further explore the role of the *ANXA1* gene in clinical application, we examined the prognostic value in all kinds of subtypes in gliomas. We used the quartile of *ANXA1* expression to divide the samples into three groups and explore their prognostic differences (**Dataset 1**). Gliomas with high expression levels of *ANXA1* showed a significant poor prognosis for overall survival (OS) in both gliomas and LGGs (log-rank test, *p* < 1.0e-4, Figures 6A,B). We also found that *ANXA1* expression stratified patients with *MGMT* promoter methylation into distinct survival groups (log-rank test, *p* < 1.0e-4, Figure 6C), assuming that patients previously thought to be sensitive to TMZ could be stratified based on *ANXA1* expression. Meanwhile, our results suggest that patients previously thought to be resistant to TMZ can be stratified based on *ANXA1* expression, and patients with low *ANXA1* expression could also have a good prognosis (log-rank test, *p* < 1.0e-4, Figure 6D). These analyses in **Dataset 2** were conducted in parallel (log-rank test, all *p* < 1.0e-4, Figures 6E–H). Furthermore, we conducted the univariate and multivariable Cox regression analyses in **Dataset 1**, which implies that *ANXA1* expression is an independent predictor for survival prognosis after adjusting for other clinicopathological factors (Table 2). These results suggest that *ANXA1* expression is significantly correlated with patient outcome.

**TABLE 2:** Univariate and multivariate analysis of clinical prognostic parameters in **Dataset 1**.

Variable	Univariate analysis			Multivariate analysis		
	*HR*	95% *CI*	*p-*value	*HR*	95% *CI*	*p*-value
WHO III	3.498	2.287 ∼ 5.348	<0.0001	3.705	2.329 ∼ 5.893	<0.0001
WHO IV	8.902	5.996 ∼ 13.215	<0.0001	6.814	4.259 ∼ 10.903	<0.0001
Gender (male)	0.924	0.702 ∼ 1.216	0.572	—	—	—
Age of diagnosis	1.033	1.020 ∼ 1.046	<0.0001	1.010	0.998 ∼ 1.024	0.096
*IDH* status (wild type)	2.777	2.099 ∼ 3.674	<0.0001	0.851	0.588 ∼ 1.232	0.393
1p/19q co-deletion status	5.887	3.608 ∼ 9.606	<0.0001	3.279	1.918 ∼ 5.603	<0.0001
*MGMT* promoter methylation status	1.196	0.909 ∼ 1.573	0.202	—	—	—
Chemotherapy (without therapy)	0.686	0.511 ∼ 0.922	<0.050	1.452	1.048 ∼ 2.013	<0.05
Radiotherapy (without therapy)	1.571	1.134 ∼ 2.176	<0.01	1.286	0.908 ∼ 1.821	0.157
*ANXA1*	1.002	1.002 ∼ 1.003	<0.0001	1.002	1.000 ∼ 1.002	<0.005

## Discussion

Since the advanced therapeutic classical model including surgery followed by commitment radiotherapy and chemotherapy with temozolomide, the median survival time remains poor with 14–16 months for recent 10 years (Stupp et al., 2005). The discovery of the lymphatic system in the central nervous system proposed a new theoretical basis and reformed the past view regarding the immunotherapy for brain tumors (Louveau et al., 2015). Therefore, more effective treatment methods were needed to improve survival in these patients.

Annexin A1 (ANXA1), also known as lipocortin I, is a Ca2^+^-dependent phospholipid-binding protein (Rescher and Gerke, 2004). It not only plays a regulated role in the process of inflammation and immunity (Perretti and D'Acquisto, 2009) but also is deregulated in multiple cancers, where it may participate in tumor development and metastasis, as summarized in previous reports (Foo et al., 2019; Bai et al., 2020). To explore the exhaustive function of *ANXA1* in gliomas, we integrated the bulk genomic and transcriptomic profiles and scRNA-seq data to comprehensively characterize the role of *ANXA1* in gliomas. In this study, we revealed that ANXA1 was significantly upregulated in GBM patients, especially enriched in *IDH* wild-type gliomas, which was consistent with previous reports (Lin et al., 2021; Qiu et al., 2020). In addition, gliomas with chemotherapy and/or radiotherapy tend to have a high *ANXA1* expression. From the somatic mutation and copy number alteration data, we confirmed that glioma-related mutations in *TP53*, *ATRX*, *EGFR*, *PDGFRA*, and others previously not recognized, including *RYR2*, *IGSF10*, *BNC2*, *CADPS2*, *COL12A1*, *TRABD2A*, and *USP34*, were significantly enriched in the higher *ANXA1* expression group, while the deletion in *CDKNA2A/B* correlated with higher *ANXA1* expression. As observed from previous reports of *ANXA1* in different cancers (Bai et al., 2020), we also revealed that *ANXA1* was mainly involved in immune-related functions, such as interferon-gamma response and regulation of innate immune response. Notably, in single-cell–level analysis, we validated that *ANXA1* exhibited a significant positive correlation with the expression of M2 macrophages and was significantly overexpressed in MVP enriched in the MES TCGA subtype. Importantly, in our analysis from 1018 CGGA samples, higher *ANXA1* expression predicted a poor prognosis in gliomas.

Despite increasing studies postulating the roles of *ANXA1* in cancer, the consensus holds that *ANXA1* in cancer cells might only be a partial functional mediator of tumorigenesis and metastasis, so it does not simply qualify as a tissue-specific mediator for predicting the occurrence of metastasis or cancer in general, due to its differential expression between different cancers. In gliomas, although there had been several reports confirming the overexpression of *ANXA1* and that it may be a prognostic and immune microenvironmental marker, the exhaustive functions of *ANXA1* in gliomas remain unclear. Consistent with previous results, we further validated that *ANXA1* was mainly upregulated in MES gliomas and macrophages, especially overexpressed in the pseudopalisading cells around the necrosis and microvascular proliferation region which further precisely confirmed the location of *ANXA1*, indicating that *ANXA1* could drive transitions to MES-like states in gliomas and plays an important role in M2 macrophages to induce the inhibitory glioma microenvironment. The details in moving the interaction of tumor cells and macrophages in gliomas will be our next study focus.

*ANXA1* has also been shown to affect the sensitivity of cancer cells to various chemotherapeutic drugs. For instance, the silencing of *ANXA1* with specific targeting compounds could increase cisplatin sensitivity to drug-resistant A549 cells (Wang et al., 2010). In our study, we also found that *ANXA1* was highly expressed in recurrent GBMs, and patients with *MGMT* promoter methylation possessed a lower *ANXA1* expression level in GBMs. As we know, the *MGMT* promoter methylated status has a confirmed association with TMZ therapy in GBMs; thus, we imply that *ANXA1* not only functions as an important factor of the post-surgery recurrence of glioma but also results in the resistance of TMZ chemotherapy. In the far future, the combined strategy of TMZ and anti-ANXA1 may improve the prognosis of GBMs.

In our current study, we elaborated the functions of *ANXA1* in gliomas from different datasets, including gene mutations, CNAs, and transcriptomic RNA sequences, especially at the single-cell transcriptomic level. Compared with the previous studies, we revealed that *ANXA1* was also upregulated in M2 macrophages derived from the glioma immune microenvironment, indicating that *ANXA1* may exert pro-tumor and inhibitory immune effects in both tumors intrinsically and the tumor microenvironment. Additionally, inhibiting *ANXA1* would decrease post-surgery recurrence or relapse of GBMs and prolong patients’ survival times. In summary, these findings have proposed that *ANXA1*, a key gene in glioma, in moving the tumor cell and glioma inhibitory microenvironment, can be a promising direction for the therapeutic strategy in gliomas. The further mechanism and intervention treatment require extensive studies to validate *in vivo*. We hope that these results would provide a new insight into future diagnosis and therapy in gliomas.

## Data Availability

The datasets presented in this study can be found in online repositories. The names of the repository/repositories and accession number(s) can be found in the article/Supplementary material.
